# Songs for the Ego: Theorizing Musical Self-Enhancement

**DOI:** 10.3389/fpsyg.2016.00002

**Published:** 2016-01-20

**Authors:** Paul Elvers

**Affiliations:** Department of Music, Max Planck Institute for Empirical AestheticsFrankfurt, Germany

**Keywords:** music and affectivity, self-enhancement, self-esteem, music, music and social cognition, music and emotion

## Abstract

This paper outlines a theoretical account of musical self-enhancement. I claim that listening to music serves as a resource for actively manipulating affective states so that a positive self-view is maintained and a sense of optimism is provided. Self-enhancement—the process by which individuals modify their self-worth and gain self-esteem—typically takes place in social interactions. I argue that *experiencing music* may serve as a unique “esthetic surrogate” for interaction, which equally enables self-enhancement. This ability relies on three main characteristics of the musical experience, namely, its capacity to (a) evoke empathetic feelings, (b) elicit social cohesion and affiliation, and (c) elicit feelings of reward. I outline how these characteristics relate to theories of music cognition and empirical findings in psychology and neuroscience research. I also explain the specifics of musical self-enhancement and how it differs from music’s other regulatory functions such as mood- and emotion regulation. My aim in introducing the notion of musical self-enhancement is to broaden our understanding of how music functions as an environmental resource entailing access to unique affective states and how musical experiences are co-constituted by both the agent and the sonic environment. This specific use of music for self-enhancement can be regarded as a form of affective niche construction, providing the external conditions in which people can experience themselves more positively and maintain high self-esteem.

## Introduction

Historical and empirical evidence supports the claim that music has a profound capacity to evoke positive feelings and pleasure in the listener. The variety of feeling states ranges from intense life-changing experiences (Gabrielsson, 2011) to subtle mood changes in everyday life (Hargreaves and North, 1999). A large body of research has also emphasized the role of music in self- and identity processes along with its importance for self-development (Larson, 1995; Ruud, 1997; DeNora, 1999; North and Hargreaves, 1999), social identity (Tarrant et al., 2001; Shepherd and Sigg, 2015), personality (Rentfrow and Gosling, 2003), and interpersonal perception (Rentfrow and Gosling, 2006). The omnipresence of music owing to current technological developments such as streaming services and portable music devices makes music highly accessible and personalized. These advances have enabled listeners in various environments and situations to make use of music’s functions, such as regulating energy and arousal levels and changing mood states (Saarikallio, 2010), as well as reinforcing self-identity (DeNora, 1999). It has also been suggested that listening to music functions as a form of empowerment (Batt-Rawden et al., 2005).

In today’s music, the topic of empowerment has become particularly apparent in hip-hop culture. Rap music has been credited with a profound capacity to voice reality and to form a meaningful identity for the urban communities in which it originates, and thus it may serve as empowerment at the individual and community levels (Travis, 2012). But empowerment also plays a role in our everyday encounters with esthetic objects in general. According to Maksimainen and Saarikallio (2015), the feeling of empowerment is among the three most intensely experienced emotions arising from encounters with music and art in everyday life.

However, accounts of the empowering potential of music can be found throughout music history and traced back as far as ancient Greece. Plato’s *Republic* gives us one of the first accounts of music as reaching far into the dimensions of agency and the self by equipping individuals with the attitudes and characteristics they need to cope with the challenges of everyday life. In the *Republic*, the Dorian mode is described as the “harmony that would fittingly imitate the utterances and accents of a brave man who is engaged in warfare or in any enforced business, and who, when he has failed […] confronts fortune with steadfast endurance and repels her strokes” (*Rep*. 3.399a5–c4, Woerther, 2008, p. 91). Ethnomusicologists have also documented various ways in which ritualized music and dance performances serve to display prestige, strength, and power (Dissanayake, 2006). The Maori battle cry “Haka” (World Rugby, 2015) that is regularly performed by New Zealand sport teams prior to international competitions provides a lively example of how song and dance are used both as a display of power, fitness, and strength, and also as a means to put the performers themselves in the right mindset.

In a recent article, journalist Romano (2013) proposed the rise of “selfie-pop,” a new trend in popular music. According to Romano, today’s pop stars, like Rihanna, Lady Gaga, Katy Parry, Ke$ha, Pink, and the Black Eyed Peas, are increasingly drawn to present themselves in their songs as strong, powerful, self-confident, and self-loving persons. The songs are not only extremely positive self-portrayals but also “songs that are all about *me*, the singer, and/or you, the listener, and how we’re going to overcome every obstacle we encounter because we’re strong and beautiful and unique and empowered and we have really great self-esteem” (Romano, 2013). This observation clearly shows that the topic of empowerment has reached the core of today’s popular music culture. But with regard to the considerable success of these kinds of songs, the question arises: Does listening to them in fact elevate self-esteem and bolster self-worth and, if it does, what psychological mechanisms might account for these phenomena?

While empowerment can occur at both the collective and the individual level and may involve different aspects such as self-esteem, resilience, growth, and change (Travis, 2012), this paper is primarily concerned with empowerment at the individual level. This is understood as how self-evaluative processes are altered, positive self-views are facilitated, and self-esteem is promoted. While music’s ability to evoke emotions has been a subject of considerable interest over the past two decades (cf. Juslin and Sloboda, 2010), the way in which listening to music helps individuals maintain positive self-evaluations and improve their self-esteem has received little attention. Few researchers have attempted to use measurements other than those related to mood and emotion to assess the feeling states of people who listen to music. One exception can be found in the work of Brown and Mankowski (1993), who showed that music-induced mood changes affect self-evaluations. The authors found that people who had listened to happy music evaluated themselves more positively than people who had listened to sad music. Interestingly, the changes in self-evaluation were more pronounced in listeners who reported low self-esteem. In particular, after listening to sad music, listeners with low self-esteem evaluated themselves more negatively than listeners with high self-esteem. Recent empirical evidence corroborates the notion that listening to music momentarily affects self-esteem (Elvers et al., 2015). These authors found that after listening to empowering pop songs that were perceived as motivating and expressive of positive self-views, people reported significantly higher levels of state self-esteem. In contrast to the findings of Brown and Mankowski (1993), this effect was not accompanied by changes in mood.

Other research has pointed out that listening to music may elicit feelings of power, make people feel more powerful, and implicitly activate the notion of power (Hsu et al., 2014). Musical agency – i.e., the intentional modulation of sounds – can also reduce perceived exertion during strenuous physical performance (Fritz et al., 2013), suggesting that musical activities exert a positive effect on physical exercise. The use of music by athletes prior to important competitions clearly demonstrates music’s potential to empower. Athletes need to be at the peak of their self-confidence, and the correct use of music might contribute greatly to this end. In fact, sport psychologists have already discussed the potential usefulness of music in the context of applied performance enhancement (Terry and Karageorghis, 2011).

But music also helps to alleviate anxiety, which, in turn, has been shown to be negatively correlated with self-esteem (Brockner, 1984). A recent meta-analysis regarding the use of music for postoperative recovery after medical surgery shows that listening to music reduces postoperative pain and anxiety and increases patient satisfaction (Hole et al., 2015). The authors suggest that music is a safe, non-invasive, and inexpensive intervention that may have sustainable positive effects on postoperative recovery. Music’s ability to reduce anxiety and promote optimism has also been linked to the elicitation of nostalgia (Routledge et al., 2008). Cheung et al. (2013) showed that nostalgia—which was induced by having the subjects read lyrics of self-selected nostalgic songs—promotes social connectedness that increases self-esteem, which in turn enhances optimism.

In the following sections, my aim is to disentangle the complex psychological process involved in empowering musical experiences and to offer a theoretical framework that accounts for possible ways in which listening to music may be self-enhancing. After introducing the notion of self-enhancement, I focus on three aspects of musical experiences and point out the ways they are related to self-enhancement processes. These are the capacity of music to (a) evoke empathetic feelings, (b) elicit social cohesion and affiliation, and (c) elicit feelings of reward.

## Musical Self-Enhancement: Feeding the Ego

It has been proposed elsewhere that music may serve as a resource for the regulation of mood (Saarikallio, 2010) and self (Krueger, 2014). However, the notion of musical self-enhancement introduces a novel perspective. While the reasons why people listen to music are manifold (cf. Schäfer et al., 2013), the notion of musical self-enhancement addresses the specific use of music listening that is related to a gain in self-worth and the psychological processes that are involved.

Self-enhancement—that is, a tendency to evaluate oneself positively—is a fundamental part of human nature (Sedikides and Gregg, 2008). An extensive amount of empirical research has shown that people’s self-evaluations are often heavily positively skewed (Beer, 2012). It has been shown that people are more likely to ascribe positive outcomes to their own capacities and efforts and negative outcomes to external factors (Campbell and Sedikides, 1999) and to use positive traits rather than negative traits to describe themselves (Alicke, 1985), and also that people generally believe that they have more personal control over external factors and maintain more optimistic views about the future than are presumably justified (Taylor and Brown, 1988). This tendency toward extremely positive self-evaluations manifests itself in several psychological patterns, such as self-serving biases (Campbell and Sedikides, 1999), optimism biases (Sharot et al., 2007), and overconfidence effects (Larrick et al., 2007). However, these “positive illusions” apparently do not indicate maladaptive behavior but rather serve everyday psychological needs (Taylor and Brown, 1988) and may have an important function with respect to mental health (Taylor and Brown, 1988; Taylor et al., 2003).

The main aim of self-enhancement is to maintain and bolster self-worth and self-esteem (Beer, 2012). While its exact meaning varies, self-esteem generally describes the evaluative part of the self-system. It can be understood as a *global* judgment of self-worth (Rosenberg, 1965), as *domain specific*, such as in sports or physical appearance, or it can be defined in relation to *self-competence* (James, 1890/1983). Self-esteem can be conceptualized both as a *dispositional trait* that is more and less stable over time, or as a *state*, which is fluctuating and moment specific (Heatherton and Polivy, 1991).

In the account proposed here, self-esteem is understood in the most basic sense, as the global evaluation of self-worth. According to a framework proposed by Crocker and Wolfe (2001) every person has an individual level of trait self-esteem that can be considered a baseline around which different levels of state self-esteem fluctuate in response to external circumstances and momentary events. Self-esteem plays an important role in well-being and happiness. It has been found that high self-esteem is related to less anxiety (Brockner, 1984), fewer depressive symptoms (Tennen and Herzberger, 1987), less hopelessness (Crocker et al., 1994), and greater life satisfaction (Myers and Diener, 1995) than low self-esteem.

The concept of musical self-enhancement is compatible with Krueger’s (2014) proposal of a “musically extended mind.” It shares the assumption that music is an important vehicle granting access to novel emotional experiences. Krueger (2014) has in detail pointed out how music listening can be understood as a distributed cognitive process. He also pointed out how the body, via entrainment and elicitation of movement, plays an important role in the realization of it. These aspects will not be discussed in more detail here. However, while Krueger (2014) also mentions that these experiences may serve self- and emotion-regulation purposes, he does not address the experiential components in greater detail. On the one hand self-regulation encompasses a variety of diverse processes that may involve changing one’s moods, maintenance of self-control and inhibition of temptation (Baumeister and Vohs, 2012). On the other hand emotion-regulation also encompasses a variety of processes such as attentional deployment and cognitive reappraisal (Gross, 2008). It also appears that some music emotion-regulation strategies such as rumination are maladaptive (Carlson et al., 2015).

The framework proposed here does not offer an exhaustive explanation for the experiential states elicited by music listening but rather considers in detail a specific subset of experiences that have up till now received considerably less attention in terms of a systematic understanding. It thus does not intend to replace other accounts of self-, mood-, and emotion-regulation but rather to complement them. Musical self-enhancement offers a framework for the kind of musical experiences that elicit positive affect related to the self, that induce feelings of power and control, that promote positive self-evaluations, and ultimately promote self-esteem.

Although self-esteem is positively correlated with affect (Brown and Marshall, 2001), it signifies a different construct. Musical self-enhancement therefore differs from mood-regulation with music since it does not only assume a change of the affective state of the listener, but also a change in the self-evaluative attitude, which is more associated with the cognitive domain: “Self-esteem means characterizing some parts of the self in descriptive terms” such as “power, confidence, and agency” (Mruk, 2006, p. 10). The concept of musical self-enhancement therefore allows the avoidance of “a too narrow focus on the unhelpful binary divide between emotion and cognition” (Clarke, 2014, p. 364) when considering the experiential dimension of listening to empowering music.

Sedikides and Gregg (2008) distinguish four levels of self-enhancement. The term can refer to either (1) the *underlying motive* that describes people’s striving for positive self-evaluations or (2) the *personality trait*, describing the habitual presence of self-enhancement. It can also describe (3) an *ongoing process*, that is, the actual realization of the motive, or (4) the *outcome* of this process. The notion of musical self-enhancement refers primarily to the third level of self-enhancement, because it signifies how the ongoing process of listening to music bolsters the listeners’ self-evaluation. A closer look at different aspects of the musical experience will illuminate how listening to music comes into play in self-enhancement processes.

## Empathy and Musical Subjectivity: Ego Projection

A very intuitive way to think of listening to music as self-enhancement is the following: When listening to an empowering song in which the singer expresses a positive self-view, the listener *empathizes* with the singer, in a way imagines *being* the singer, and through this process adopts and projects the expressed self-view, at least to a certain extent, onto her- or himself.

Since the early stages of modern esthetics, this kind of empathetic perspective taking has been proposed as a potential mechanism that can explain how people perceive esthetic objects. Vischer (1887) introduced the notion of *Einfühlung* (“feeling in”) to explain how the perceiver of an esthetic object gains access to the psychological depths of the artwork and its inner life. Lipps (1906) then developed the notion to account for the more general social cognitive process of sharing the feelings of other human beings. The basic mode of empathy that is related to the German notion of *Einfühlung* has been characterized as “an *experiential* access to the other’s subjectivity” (Colombetti, 2014, pp.173–174). This empathetic state is comparable to our state when we re-experience memories from the past. The experiences of others as well as of ourselves in past experiences happen in a specific way that has been described as “non-primordial” (Colombetti, 2014, chap. 7). That is, although the observed or imagined feeling state is not experienced “as one’s own,” it is still experienced directly. *Basic empathy* with another’s feeling state is experienced in the same way as the joy in remembering a joyful event, in a direct non-primordial way.

Given that music listening may be conceptualized as a *special* form of social communicative processes (Cross, 2011), the assumption that empathy plays an important role lies at hand. Empathy in music listening has been a recent subject of considerable interest and has been examined from various perspectives (cf. Clarke et al., 2015). It has been shown that dispositional empathy is associated with music-induced sadness (Vuoskoski and Eerola, 2012) and that the discrepancy between *felt* and *perceived* emotions (Gabrielsson, 2002) can be reduced when empathetic reactions to the interpreter/performer are stronger (Egermann and McAdams, 2013). Empathy has also been proposed as a potential mechanism explaining how music induces emotions in general (Scherer and Zentner, 2001; Koelsch, 2013; Clarke, 2014).

These conceptions center on the idea that when listening to music, people imagine the feeling state of *either* the person responsible for making the music (i.e., the composer/performer) or an *imagined* or *virtual persona* (Cone, 1982; Robinson, 2005; Robinson and Hatten, 2012) to whom they ascribe the feeling state. Levinson (2006, p. 115) argues that it is a fundamental aspect of musical expressivity that we “construe it as if it is or harbors an *individual externalizing its inner life.*” This means that when we perceive music as being (emotionally) expressive, we tacitly assume that a “musical subjectivity” (Kramer, 2001) is at stake. One might think of many examples that would challenge such a conception of music listening, for example, when listening attentively to abstract instrumental music or contemporary noise music. In such cases, we do not necessarily assume that people think of the music as an expression of a *person.* Some authors (e.g., Cone, 1982) believe the persona to be necessarily involved when music is perceived as expressive. Since music is always the product of musicians, and therefore always the product of human agency, we can approach any artwork with the “background belief that it has been deliberately constructed by a human being” (Cochrane, 2009, p. 204), so that imagining a musical persona might be an automatic and unconscious process. However, the degree to which a musical piece is perceived as expressive of a musical subjectivity presumably varies between (sub-)cultures and musical styles. An empathetic mode of listening may be more or less pronounced depending on the degree to which the music is intended to be perceived as such (Wald-Fuhrmann, 2013). Nevertheless, compared to challenging cases of music where the degree of perceived subjectivity might be lower, the concept of an empathetic mode of listening seems promising for the case of empowering pop songs, since here the singers are perfect candidates for the personae, and in their iconic role, today’s pop stars can clearly be seen as objects of empathetic identification.

While the cognitive and affective states of others are simulated in one’s own mind in empathetic reactions, empathy usually implies that a self–other distinction is maintained in these experiences (Coplan, 2011). This means that empathetic reactions to music or a musician do not necessarily imply identification or a blending of self and other. However, various accounts show that in many cases, music is perceived in a way that suggests some sort of self/other blending between the musical persona and the listener (cf. Clarke, 2014). For example, in their qualitative study, Greasley and Lamont (2011) describe how one of their participants listens to a song on a daily basis because it helps her to find a sense of identity and to understand herself better. She perceives the songs of her favorite band as “very original, deeper and more honest than any I’ve heard before, *they say things that I feel* and daren’t express to anyone else” (Greasley and Lamont, 2011, p. 60). In this intense listening episode, the music reveals something of her own personality to the listener and is in a way perceived as being self-expressive and self-confirming. Another example can be found in Gabrielsson’s collection of qualitative descriptions of strong experiences with music. Here, a young woman describes her experience of a live flamenco guitar performance in the following way: “It felt like…*it agreed with me exactly, my inner self*. I still feel like that with flamenco—*it is exactly as if it expresses what I feel inside, my soul, my feelings*” (Gabrielsson, 2011, p. 202).

Such empathetic reactions in music listening may be regarded as different compared to those in regular social interaction since they may involve a blending of self and other. When music is perceived as self-expressive and self-confirming, listeners experience the musical subjectivities as part of their own self. They may be best described with Cohen’s (2001) account of identification – an intense empathetic reaction in media recipients. Cohen (2001) assumes that the distinction between self and other is blurred, “because when identifying, one lacks an awareness of the self, and, therefore, the distinction between self and other” (Cohen, 2001, p. 253). This may be regarded as one of the unique features of music that serves as an *esthetic surrogate* of social interaction. It enables empathetic processes but allows the listeners to lose themselves in the music and identify themselves with the musical persona. Empathetic reactions in esthetic contexts have the unique feature that they happen in a “safe environment.” When appreciating esthetic objects, people may engage in social cognitive processes but usually in a “disinterested” or “dissociated” manner, meaning that “in the case of music […], the listener will know at an automatic, subconscious level that the events cannot cause harm to him or her” (Schubert, 2009, p. 70). While a musical persona may trigger empathetic processes, it is not an *active subject* participating in the interaction itself, which is why it may lead to reduced self-control and self-awareness in the listener and an intensified identification with the persona.

According to Cochrane (2009), an empathetic listening mode can be explained by simulation theory, a theory about how we understand others that has generated considerable interest from both philosophers and neuroscientists (Gallese and Goldman, 1998; Goldman, 2006; Grafton, 2009). Simulation theory assumes that we gain information about other minds by putting ourselves in their “mental shoes,” thus, simulating what others feel or think also involves projecting ourselves into the position of the other (Zahavi, 2008). Cochrane (2009) argues that music may “hijack” the simulation mechanism that is usually involved in detecting emotional states of the self and others (Goldman, 2006). When listening to music, we mirror the movements of the music from a first-person perspective, which—when affectively expressive— will arouse our emotions (Overy and Molnar-Szakacs, 2009).

Simulation theory has been criticized on various grounds that cannot be fully addressed in this paper (Zahavi, 2008; cf. Colombetti, 2014, chap. 7). However, the fact that simulation processes may operate unconsciously (Currie, 2011) may weaken one of the objections to simulation theory, namely, that simulation plus projection is an overly complex process that does not meet the phenomenologically direct and immediate experience of empathy. That is, it might be the case that, while listening to music, people experience emotions that were evoked by simulating another mental state (that of the musical agent), but they might not be aware of simulating another’s mind if this mechanism is operating unconsciously.

The empathetic listening mode provides a potential explanation for musical self-enhancement. Music may provide an easy and accessible environmental resource that allows self-worth and self-esteem to be generated by empathizing and identifying with a musical persona who shares positive attributes and a high sense of self-worth. Specific musical subjectivities that are characterized by a confident, strong, and positive self-view may provide the “mental scripts” (Colombetti, 2014, p. 46) that enable episodes of empowerment. The music provides virtual subjectivities that are explored, and eventually, to a certain extent, adopted. Since it has been shown that people holding a positive self-image in mind report higher levels of self-esteem than those with negative self-images (Hulme et al., 2012), an empathetic process in which a positive self-view of the musical persona is to a certain extent transferred onto the listener presumably enables the self-enhancing effect of empowering music.

However, this effect may crucially depend on the listeners’ susceptibility to engagement in the empathetic process. This susceptibility may be accounted for by individual differences such as dispositional empathy (Clarke et al., 2015) or the musical taste of the listener, and situational factors. It is therefore important to point out that musical self-enhancement is a culturally established practice where, on the one hand composers, musicians, and music producers essentially draw on music’s ability to provide access to feeling states in which self-worth is high, and on the other hand, listeners share, at least to a certain degree, a familiarity with and appreciation of this particular type of music, enabling them to benefit from its self-enhancing function.

## Social Cohesion and Affiliation

Another key function of music making and listening that has been discussed in relation to its use today as well as its evolutionary development concerns its capacity to create and strengthen social bonds among those who interact and engage in musical activities (cf. Tarr et al., 2014). Ample empirical evidence corroborates the notion that shared musical tastes and preferences can create social identities (Tarrant et al., 2001; Tekman and Hortacsu, 2002; Lonsdale and North, 2009) and bonding (Boer et al., 2011). Self-report data reveal that social functions are considered to be among the most important functions of music listening (Hargreaves and North, 1999; Boer et al., 2012; Schäfer et al., 2013). In addition, music making and music listening promote prosocial behavior (Greitemeyer, 2009; Kirschner and Tomasello, 2010). Scholars from different academic backgrounds have emphasized that the social function of music lies at the core of how it developed among humans. Music ethnologists (Merriam, 1964) and anthropologists (Suppan, 1984, 1986) have argued that music fulfills various social functions in a number of areas spread wide around the world. Empirical research by the social psychologists Loersch and Arbuckle (2013) has corroborated this notion by showing that musical reactivity (i.e., the tendency to be affected by musical information) is related to other social motivations, which suggests that interaction with music fulfills social needs.

It has been emphasized that synchronization facilitates a merging of self and other. It may also strengthen social bonds between those who synchronize. Abundant evidence shows that synchronization enhances the positive evaluation of interaction partners, even in cases where people synchronize solely on the basis of auditory perceptual information (cf. Tarr et al., 2014). That musical experiences entail synchronization may be explained by the fact that people are extremely susceptible to entraining to musical rhythms (Clayton et al., 2005) or—in other words—because “music affords movement” (Krueger, 2014, p. 2). However, the idea of human agency may also play an important role in synchronization processes. In the case of private listening experiences, imagining the musical agent (i.e., the musician or persona) for whom the music is a communicative expression provides an interaction partner to synchronize with, which would presumably be lacking if the perceived sound were mere noise. Indeed, synchronization is facilitated among children in joint music-making episodes when they interact with other human beings instead of drum computers (Kirschner and Tomasello, 2009).

Another perspective on the social functions of music listening draws on the fact that musical agents can also be perceived as having an empathetic reaction to the listener. Just like a good friend, musical experiences have sometimes been described as if the musical agent were understanding and compassionate. Van den Tol (2011) explored the reasons people listen to sad music when they feel sad. Participants in this study described “the experience of listening to sad music like being with a good friend, or suggested the music to have characteristics of a friend” (Van den Tol, 2011, p. 453). Saarikallio and Erkkilä (2007) provided a similar account, reporting that one of the reasons why adolescents use music to regulate mood states is that they experience solace through listening to music. Solace is another emotion that suggests an empathetic reaction to the music or the musical agent by the listener. Additionally, Lee et al. (2013) corroborated the notion that music may serve as an “esthetic surrogate” for social interaction by showing that study participants sought mood-congruent musical stimuli and that musical preferences correlate with preferences for an empathetic friend.

Contemporary psychological theories of self-esteem, such as sociometer theory (ST; Leary and Downs, 1995; Leary, 2005) and terror management theory (TMT; Greenberg et al., 1986; Pyszczynski et al., 2004), claim that self-esteem is strongly tied to a person’s social standing. While TMT maintains that self-esteem is mainly fueled by the beliefs that (a) one is living in a culturally meaningful universe and (b) one is living up to one’s own standards and is thus a proper member of the valued culture, ST claims that self-esteem essentially relies on interpersonal acceptance and the individual’s relational value (Leary, 2005). According to ST, self-esteem functions like a gage that indicates one’s social acceptance, with the self-esteem system forming a complex set of processes that work to maintain the social acceptance. The output of the system is what traditionally has been conceptualized as self-esteem (i.e., self-worth), which responds directly to social interactions by monitoring one’s social acceptance and helping to respond to potential threats. Empirical evidence corroborates the notion that social feedback is among the most effective strategies for manipulating self-esteem (Heatherton and Polivy, 1991; Leary, 2005).

The TMT perspective, which emphasizes that the reinforcement of a meaningful cultural worldview fuels self-esteem, can be accounted for by the fact that people often describe experiencing the music they like as something intensely meaningful: “We all hear the music we like as something special, as something that defies the mundane, takes us ‘out of ourselves,’ puts us somewhere else” (Frith, 1996, p. 275). Within the TMT framework, Kneer and Rieger (2015) showed that listening to heavy metal music helps heavy metal fans overcome the fear of death and leads to increased identification with the heavy metal in-group. Apart from TMT framework’s theoretical assumption that reduction of the fear of death primarily relies on the activation of a salient *cultural worldview*, the authors suggest that the effect might also rely on self-enhancement processes and thus on bolstering self-esteem (Kneer and Rieger, 2015).

In light of these theoretical considerations, the social connectedness that arises from musical experiences might be a driving factor in musical self-enhancement. So another way to think about how music can enhance self-esteem is the following: In and through music, social affiliation is experienced. The music might be experienced like a “good friend,” it might signify a particular social identity, or it might just happen that a feeling of connectedness arises at the basic level of synchronized movements. The social connectedness in turn may enhance our relational value and interpersonal acceptance and thus enhance our self-esteem. A similar relationship has been experimentally observed when the feeling of nostalgia is induced. Cheung et al. (2013) found that reading nostalgic texts increased social connectedness, which in turn enhanced self-esteem.

## Pleasure, Positive Affect, and the Self

The third aspect that comes into play in musical self-enhancement processes is pleasure. The fundamental assumption that music evokes pleasure in both the performer and the listener has been documented at least since antiquity, and accounts of it can be found in both Plato (*Laws* 5.732e4–7) and Aristotle (*Politics* 1337b23–36) (Plato, 1994; Aristotle, 1944). In fact, pleasure was believed to be the key component in the education of the soul through music, as taking pleasure in virtuous kinds of musical tunes allowed young people to achieve an irrational kind of virtue: pursuing the morally right thing before having fully acquired the capacity to judge for themselves what is good or bad (Woerther, 2008).

Today, as well, most people experience music as one of the most potent sources of pleasure (Dubé and Le Bel, 2003). It has been claimed that the capacity to evoke pleasure might be the fundamental function of listening to music, while other functions are subsidiary (Schubert, 2009). Intensely pleasurable responses to music have attracted considerable interest in the neurosciences, where the focus is mainly on particularly moving “peak” experiences called *chills* or *frissons* (Panksepp, 1995). Blood and Zatorre (2001) found decreased activity in brain regions that are associated with anxiety when subjects experienced music as intensely pleasurable. They also found that peak experiences with music activate brain regions that are associated with reward. Building on this, further research on the role of dopaminergic activity in the mesolimbic system has shown that intensely pleasurable music can lead to dopamine release in the striatal system, suggesting that music may activate a neural network in a similar manner to other rewarding stimuli such as food, psychoactive drugs, and money (Salimpoor et al., 2011).

Nevertheless, the role of reward in musical self-enhancement is ambiguous and not all experiences of pleasure in music are expected to contribute to musical self-enhancement processes. Reward is usually accompanied by hedonic feeling states and reinforcing feelings of having obtained the rewarding object (Chanda and Levitin, 2013), which suggests that pleasure leads to positive affect, which in turn has a positive effect on self-esteem. But pleasure may not always induce positive affect. Indeed, it has been shown that people with a tendency toward depression reported listening to sad music as pleasurable, even though it evoked negative affect and increased feelings of depression in the listener (Garrido and Schubert, 2015).

Pleasure often has an intensifying effect on music-evoked feelings. Liking of music was found to predict the intensity of music-induced emotions (Egermann and McAdams, 2013). Liking was also found to be the strongest predictor of a self-esteem enhancing effect of music listening (Elvers et al., 2015). Regardless of whether the music was perceived as positive or negative, in these study participants reported an increase in self-esteem after listening to music when they also reported higher degrees of liking it. It may thus be expected that pleasure usually contributes to musical self-enhancement, although there may be specific conditions, such as taking pleasure in sad music among people with tendencies to depression, in which pleasure may be opposed to self-enhancement.

Pleasure might be more closely related to musical self-enhancement in situations where pleasurable responses to esthetic objects involve self-referential cognitive processes. A study that used fMRI methods to inquire about intensely pleasurable responses to esthetic stimuli found increased activity in brain regions that are part of the default mode network (DMN; Vessel et al., 2013). The brain regions involved in the DMN are particularly active when self-referential and self-relevant information is processed.^1^ Vessel et al.’s (2013) study recorded brain activation in response to artworks. Participants indicated how moving they felt the artwork was on a scale ranging from 1 (lowest) to 4 (highest). For the subset of responses that were rated as highly moving, the experimenters recorded high activity in the DMN region, suggesting that self-referential and self-relevant information is processed in esthetic experiences that are considered highly moving. Although these responses were evoked by artworks in this study, they were not correlated with any specific features of the artworks but instead with the specific esthetic response pattern of being highly moved. This suggests that they may also arise from encounters with esthetic objects other than artworks. In addition, similar response patterns and activation of brain areas involved in the DMN have been observed when subjects listen to music (for a review, see Omigie, 2015).

The picture emerging from these findings is that, at least in some cases, highly moving esthetic experiences show a greater involvement of self-relevant information processing and may elicit stronger self-referential emotional states. The experimenters compare the observed responses to self-referential emotions such as pride, shame, or guilt, although they lack an appraisal of self-responsibility for the event (Vessel et al., 2013). It can be concluded that pleasure might not be beneficial for musical self-enhancement *per se*, but it might play an important role in intensifying the induction of positive feelings. Pleasure might support self-enhancement but only when it is accompanied by positive affect or positive self-referential feelings. That positive affect in general, and, more specifically, self-referential emotions such as pride, are positively related to self-esteem, has previously been demonstrated (Pelham and Swann, 1989; Brown and Marshall, 2001).

## From Music Listening to Self-Enhancement

Musical self-enhancement can be described as a culturally established practice of externalizing or offloading self-enhancement processes onto the environment where the music serves as a vehicle that grants access to positive self-evaluations and heightened self-esteem. Music may be especially useful as an external resource for self-enhancement since it is ubiquitous, portable, easy to access, and relatively harmless. Listening to empowering music may help us in specific situations where our ego is under threat, to cope with stressful events, and to be mentally prepared for demanding situations.

When it comes to the question of what musical styles or pieces can be counted as empowering, the answer becomes more difficult. Empowering music is primarily defined as a specific *function* of music listening that is related to self-enhancement. However, in another sense, empowering music may signify a group of musical pieces. These pieces are not bound to any style, genre, or historical period; although today they are mostly found in contemporary popular music. The examples by Romano (2013) mentioned above might account as paradigmatic cases of these kinds of pieces. The subjectivities that emerge from these pieces usually display characteristics that are associated with a high level of self-worth, such as power, confidence, control, optimism, etc. They may be mostly related to an empathetic mode of listening and identification with the musical persona.

Other empowering musical pieces rely more on the aspect of social cohesion. Instead of displaying a high level of self-worth, these pieces may be self-enhancing by eliciting a feeling of social connectedness. The feeling of connectedness, in turn, may arise from the elicitation of a specific social identity or may be simply based on entrainment and synchronization. In cases where music serves as a *surrogate* for an empathetic friend, the emerging subjectivities may be characterized by the display of characteristics such as sympathy and solicitousness. Indeed, some empowering songs may even directly address the listener in a second-person narrative (Elvers et al., 2015).

The degree to which someone likes a musical piece may also play a key role in musical self-enhancement. Since there is great individual variability in musical taste and in the esthetic and evaluative responses to music, the question of how to define empowering music becomes even more difficult. So far, it seems safe to say that it is necessary but not sufficient to like the musical pieces that are considered as empowering. Presumably, those musical pieces that enable a combination of two or even all three outlined aspects have the greatest self-enhancing effect on the listener.

## Conclusion

The proposed framework of musical self-enhancement outlines a theoretical account of how music listening may come into play in self-enhancement processes and, further, bolster self-worth. Musical self-enhancement is explained in terms of three main aspects of musical experiences. The relevance of each aspect is supported by both conceptual and empirical evidence as outlined above. We find good initial empirical evidence that (in some cases) listening to music is related to self-regulative and self-evaluative processes, that under specific circumstances it effectively reduces anxiety, that it fosters social bonding and connectedness, that it may sometimes be seen as an empathetic friend, and that listening to empowering songs enhances state self-esteem.

**FIGURE 1:**
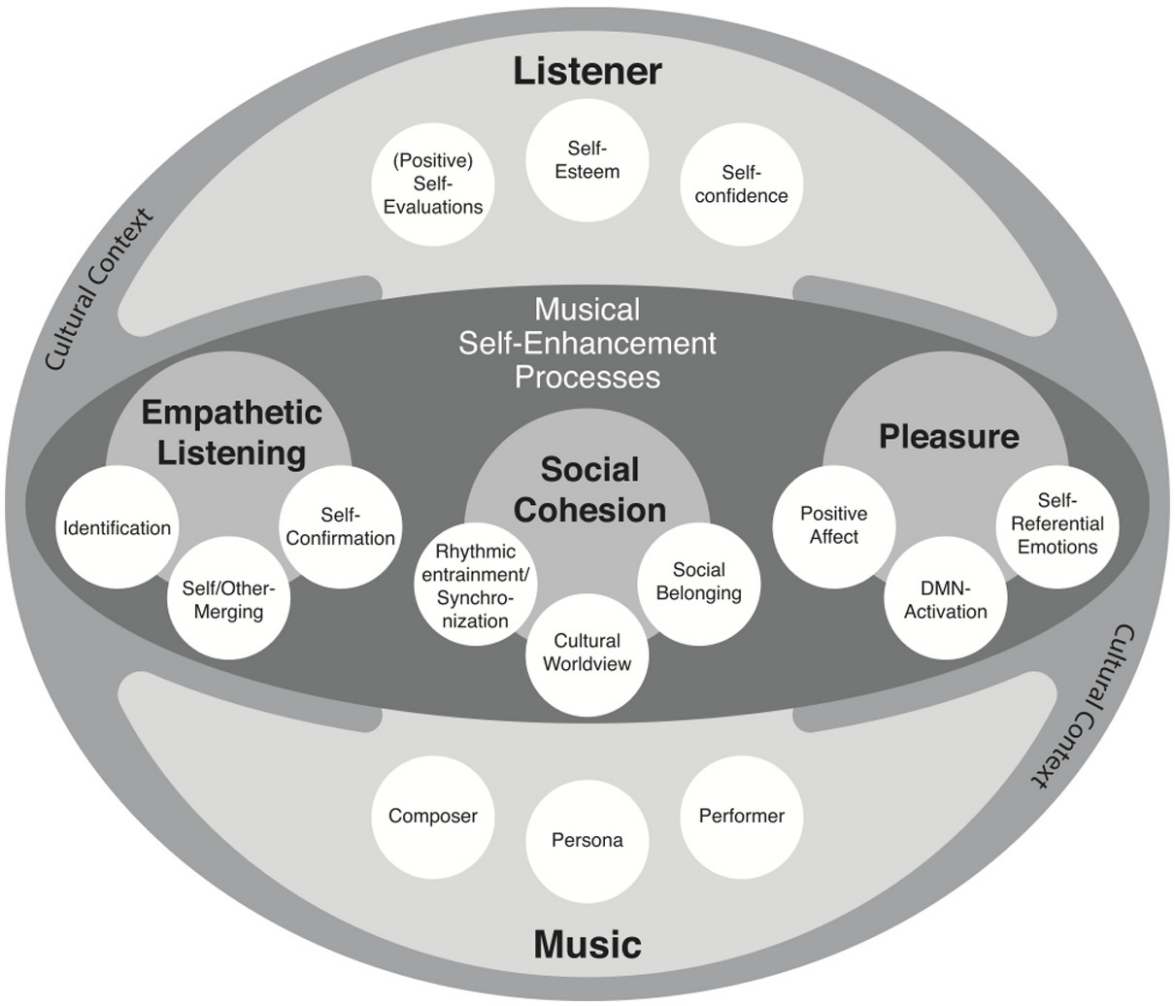
**Visualization of the framework of musical self-enhancement.** The musical experience is embedded in a cultural context. The relationship between the music and the listener is realized in terms of different aspects of listening to music that may account as potential mechanisms of its self-enhancing function.

The proposed framework highlights how external cultural artifacts such as music play an important role in people’s affective experiences in their everyday lives. It also highlights how complex cognitive and affective states may be scaffolded and (to a great extent) constituted by an external, environmental (esthetic) artifact, namely, music. In order to disentangle the complex experiential process of listening to empowering music, the three outlined aspects of musical self-enhancement provide potential mechanisms that explain the relationship between the music and the listener in musical self-enhancement processes. Each of these aspects, in turn, encompasses a variety of (sub-)processes. For instance social cohesion encompasses entrainment and synchronization, the maintenance of a cultural worldview, and social belonging. The proposed framework can therefore be understood as an initial attempt to conceptualize musical self-enhancement (see **Figure 1**).

In order to better understand the relationship between the outlined aspects of musical self-enhancement, further research should aim at disentangling empathetic listening, from social cohesion and pleasure in experimental settings. Here it would be of interest to see how each of the outlined aspects contributes to music induced changes in self-esteem. Researchers should primarily operationalize self-esteem as *state*-(Heatherton and Polivy, 1991) and *implicit* self-esteem (Greenwald et al., 2002), since these constructs are expected to be malleable by listening to music. Another approach could be to investigate music listening behavior in specific situations, such as when the ego is under threat or when one is preparing for demanding tasks, since these situations specifically demand self-enhancement processes. This approach would allow examining if the music listening behavior under these circumstances resembles other self-enhancement strategies, such as outlined by Sedikides and Gregg (2008). To further understand the role of pleasure and self-referential emotions, it would also be of interest to employ measures assessing self-referential emotions during or after listening to music, since these imply self-evaluative judgments (Brown and Marshall, 2001). This procedure would allow the establishment of a link between the field of music and emotion and musical self-enhancement.

As compared to other regulatory functions of music listening, musical self-enhancement highlights how music listening affects the *evaluative* dimension of the self. Since this dimension (i.e., self-esteem) is considered an important aspect of well-being and happiness (Crocker and Wolfe, 2001), the proposed framework provides a general explanation for the adaptive function of music listening. That being said, only scarce inferences can be drawn concerning an application of music listening as a potential therapeutic intervention. Although the proposed framework suggests that musical self-enhancement is an important function of music listening, the complex entanglement of both the characteristics of the music and the listener impede systematic considerations, and clearly more effort is needed to fully understand the processes involved. It seems to be the case that although people intuitively know how to use music as a resource for self-enhancement, the individual variability in responses to music make a generalized application of music as a treatment “like a pill” very difficult. This is why in most cases self-selected music exerts the greatest positive effects on listeners (Chanda and Levitin, 2013).

However, musical self-enhancement might provide an explanation for those cases where music clearly shows a positive effect related to self-esteem. Current research claims that music therapy does in fact help in the treatment of depression, although the exact mechanism remains uncertain (Maratos et al., 2011). Since high self-esteem has been associated with fewer depressive symptoms (Tennen and Herzberger, 1987), listening to empowering music may elevate self-esteem and thus contribute to a decrease in depressive symptoms. Here, musical self-enhancement might offer a promising explanatory framework that helps understanding of how a music-evoked enhancement of self-esteem might contribute to the treatment of psychological disorders like depression.

Even though many people have encountered the uplifting power of music in their lives and it has been documented throughout music history since Plato and Aristotle, little is known about the underlying psychological processes that enable these kinds of experiences. The outlined framework of musical self-enhancement is one attempt to approach the multifaceted phenomenon of empowering music. It will hopefully provide other scholars with food for thought and motivate more research addressing the simple but yet intricate question, of how music makes us feel better about ourselves.

## Conflict of Interest Statement

The author declares that the research was conducted in the absence of any commercial or financial relationships that could be construed as a potential conflict of interest.

## Footnotes

1 The notion that esthetic experiences entail a self-reflective component is also part of various classic and contemporary esthetic theories, e.g., Hegel’s (2008) discussion of music in his esthetic lectures or Novalis (1978) *Hymns to the Night*.
